# Comparison of Two Respiratory Support Strategies for Stabilization of Very Preterm Infants at Birth: A Matched-Pairs Analysis

**DOI:** 10.3389/fped.2019.00003

**Published:** 2019-01-29

**Authors:** Tessa Martherus, André Oberthuer, Janneke Dekker, Christoph Kirchgaessner, Nan van Geloven, Stuart B. Hooper, Angela Kribs, Arjan B. te Pas

**Affiliations:** ^1^Department of Paediatrics, Leiden University Medical Center, Leiden, Netherlands; ^2^Department of Neonatology, Children's Hospital University of Cologne, Cologne, Germany; ^3^Medical Statistics, Department of Biomedical Data Sciences, Leiden University Medical Center, Leiden, Netherlands; ^4^The Ritchie Centre, Hudson Institute of Medical Research, Melbourne, VIC, Australia; ^5^Department of Obstetrics and Gynaecology, Monash University, Melbourne, VIC, Australia

**Keywords:** birth, breathing, CPAP, preterm infants, respiratory support

## Abstract

**Objective:** Respiratory support for stabilizing very preterm infants at birth varies between centers. We retrospectively compared two strategies that involved either increasing continuous positive airway pressures (CPAP), or increasing oxygen supplementation.

**Methods:** Matched-pairs of infants (<28 weeks of gestation) were born either at the Leiden University Medical Center [low-pressure: CPAP 5–8 cmH_2_O and/or positive pressure ventilation (PPV) and fraction of inspired oxygen (FiO_2_) 0.3–1.0; *n* = 27], or at the University Hospital of Cologne (high-pressure: CPAP 12–35 cmH_2_O, no PPV and FiO_2_ 0.3–0.4; *n* = 27). Respiratory support was initiated non-invasively via facemask at both units. Infants (*n* = 54) were matched between centers for gestational age and birth weight, to compare physiological and short-term clinical outcomes.

**Results:** In the low-pressure group, 20/27 (74%) infants received 1–2 sustained inflations (20, 25 cm H_2_O) and 22/27 (81%) received PPV (1:19–3:01 min) using pressures of 25–27 cm H_2_O. Within 3 min of birth [median (IQR)], mean airway pressures [12 (6–15) vs. 19 (16–23) cmH_2_O, *p* < 0.001] and FiO_2_ [0.30 (0.28–0.31) vs. 0.22 (0.21–0.30), *p* < 0.001] were different in low- vs. high-pressure groups, respectively. SpO_2_ and heart rates were similar. After 3 min, higher FiO_2_ levels [0.62 (0.35–0.98) vs. 0.28 (0.22–0.38), *p* = 0.005] produced higher SpO_2_ levels [77 (50–92) vs. 53 (42–69)%, *p* < 0.001] in the low-pressure group, but SpO_2_/FiO_2_ and heart rates were similar. While intubation rates during admission were significantly different (70 vs. 30%, *p* = 0.013), pneumothorax rates (4 vs. 19%, *p* = 0.125) and the occurrence of spontaneous intestinal perforations (0 vs. 15%, *p* = 0.125) were similar between groups.

**Conclusion:** Infants (<28 weeks) can be supported non-invasively at birth with either higher or lower pressures and while higher-pressure support may require less oxygen, it does not eliminate the need for oxygen supplementation. Future studies need to examine the effect of high pressures and pressure titration in the delivery room.

## Introduction and Rationale

Most very preterm infants require respiratory support in the delivery room, as they are unable to adequately aerate their lungs (1–5). Historically, infants were intubated directly after birth and respiratory support was given by mechanical ventilation with pure oxygen. In recent years the focus of respiratory support has shifted toward a gentler approach, focusing on supporting spontaneous breathing and titrating the inspired oxygen content to reduce hypoxia to quickly achieve target oxygen saturation values (6–8). A non-invasive approach is now recommended that uses either continuous positive airway pressure (CPAP) and/or positive pressure ventilation (PPV), combined with ambient or a blended air/oxygen gas mixture (9–11). Yet, there is a wide diversity in clinical practice and very little data is available on the most effective approach to support preterm infants during the cardiopulmonary stabilization (12).

In preterm infants, the presence of breathing effort can often be missed during the initial evaluation, resulting in the application of PPV within the first minutes followed by CPAP once the infant is stabilized. CPAP pressures of 4–8 cm H_2_O are currently recommended in the delivery room (9, 10), although preclinical studies (13–18) have indicated that higher positive-end expiratory pressures (PEEP) may be beneficial during mechanical ventilation. Higher PEEP levels improve lung liquid clearance and lung aeration, thereby maintaining functional residual capacity (18). As such, higher CPAP levels may also improve liquid clearance and lung aeration, which may also improve pulmonary blood flow, heart rate and oxygenation and reduces the need of supplemental oxygen and PPV. On the other hand, high CPAP levels could over expand the lungs, thereby increasing the risk on pneumothoraxes (14, 15), reduce pulmonary blood flow (13, 15, 16) and breathing rate (19).

International guidelines (9–11) nowadays recommend commencing respiratory support with air or using a fraction of inspired oxygen (FiO_2_) content of 0.3, blended with air. The FiO_2_ can then be increased to achieve oxygen saturation (SpO_2_) values within the ranges depicted by the Dawson's nomogram (20). Most infants (90–100%) with very low birth weight and preterm infants require an increase in FiO_2_ to increase oxygen saturation and avoid hypoxemia (21, 22) which has dangerous consequences. Indeed, data from eight randomized clinical trials showed that infants who did not reach an SpO_2_ of 80% at 5 min after birth, were more at risk to die before hospital discharge and to develop major intraventricular hemorrhages (23). However, supplemental oxygen increases the risk of hyperoxemia (22, 24). Due to the immaturity of the anti-oxidant defense systems, supplemental oxygen can lead to an excess of free oxygen radicals causing damage in multiple organs, thereby developing e.g., bronchopulmonary dysplasia and retinopathy of prematurity (25, 26). The control of oxygen during the neonatal stabilization should therefore be handled accurately to minimize the risk of hypoxemia while avoiding hyperoxemia. Several clinical trials have compared initiation of resuscitation with low vs. high oxygen levels, with a recent large clinical trial (27) finding a higher mortality rate when initiating resuscitation with FiO_2_ 0.21, compared to FiO_2_ 1.0. However, collectively the results of these trials are inconsistent and meta-analyses (28–30) have concluded that there is insufficient data to recommend a strategy for very preterm infants.

In this retrospective matched-pairs study, we explored two respiratory support approaches using either higher oxygen or titrated CPAP. At the Leiden University Medical Center (LUMC), CPAP pressures of 5–8 cm H_2_O and/or PPV are given while FiO_2_ levels are titrated between 0.3 and 1.0. At the University Hospital of Cologne, respiratory support commences with a CPAP of 12 cm H_2_O and CPAP pressures are step-wise increased up to a maximum of 32 cm H_2_O. The FiO_2_ is usually kept between 0.3 and 0.4. The large differences in respiratory support strategies prompted us to compare the immediate effect of these two different approaches on the physiological and short-term clinical outcomes.

## Materials and Methods

We performed a retrospective matched-pairs study and included infants born between 24 0/7 and 27 6/7 weeks of gestation. Infants born between the introduction of the New Life Box respiratory function monitor (Applied Biosignals, Weener, Germany) and August 2017 were included. The monitor was introduced at the University Hospital of Cologne in August 2014 and at the LUMC in March 2014. Infants with congenital abnormalities were excluded, as well as infants who's recording could not be identified or infants with incomplete files. Infants born at the University Hospital of Cologne were matched 1:1 with infants born at the LUMC. Matching criteria were gestational age (+/– 4 days) and birth weight (+/– 25% grams). The database was scanned in chronological order and in case of multiple potential matches, the first potential match was included. Infants could not be matched in a 1:2 ratio due to an unequal distribution of gestation and birth weight.

### Study Protocols

At the LUMC (low-pressure group), 5 cm H_2_O CPAP is given initially and can be increased to 8 cm H_2_O. If the infant is apneic or bradycardic, sustained inflations (20–25 cm H_2_O, 15 s) and PPV (PIP 25 cm H_2_O, 40–60/min) are given. The fraction of inspired oxygen (FiO_2_) is initially set at 0.3 and can be adjusted step-wise up to 1.0 based on the 25th percentile of the Dawson criteria. Respiratory support was provided by the Neopuff™ T-Piece resuscitator (Neopuff Infant Resuscitator, Fisher & Paykel Healthcare Ltd., Auckland, New Zealand) via facemask (Neonatal Resuscitation Mask, Fisher & Paykel Healthcare Ltd, Auckland, New Zealand).

In the University Hospital of Cologne (high-pressure group) infants are initially supported with 12 cm H_2_O CPAP, delivered by the Benveniste Valve™ (Dameca, Löwenstein Group, Rødovre, Denmark) via facemask (Disposable Face Mask Neonate, Ambu A/S, Ballerup, Denmark). If infants are apneic or bradycardic, the flow is increased by 2L/min subsequently increasing the pressure to a maximum of 32 cm H_2_O. Sustained inflations and positive pressure ventilation are not included in the local protocol. The FiO_2_ is initiated at 0.3 and can be increased to 0.4. The delivery room management is essentially as described by Mehler et al. (31), however lower pressures and higher FiO_2_ values were used at that time.

### Study Outcomes

The primary study outcome was SpO_2_ in the first 7 min after birth. The 7 min timeframe was chosen to minimize the effect of other medical interventions. An overview of the data showed that FiO_2_ in the lower CPAP group was increased above 0.40 in median (IQR) 2.55 (2.48–3.22) min. To compare the effect of different pressure support strategies only, we analyzed the data in Phase I and II, represented before and after FiO_2_ increase.

Secondary delivery room study outcomes were heart rate, FiO_2_, mean airway pressure and the SpO_2_/FiO_2_ ratio. The SpO_2_/FiO_2_ ratio represents the gas exchange efficiency of the lungs as the surface area and the oxygen gradient mainly drive gas exchange. Apgar score at 5 min after birth, pneumothorax rate <72 h, the incidence of intubation <72 h, intraventricular hemorrhages (> grade 2) and spontaneous intestinal perforations, reflected the short-term clinical outcome. The occurrence of spontaneous intestinal perforations could be a potential effect of high pressures on gas entering the intestinal tract. Gestational age, birth weight, gender, mode of delivery and Apgar score at 1 min after birth were collected from the medical records to describe baseline characteristics.

To record SpO_2_ and heart rate, a Masimo SET pulse oximeter probe (Masimo Radical, Masimo Corporation, Irvine, California, USA) was placed around the right wrist of the infant. FiO_2_ was measured using a portable oxygen analyzer AX300-I (Teledyne Analytical Instruments, CA, USA), and the airway pressures were registered by a variable orifice flow sensor (Avea Varflex Flow Transducer, Carefusion, Yorba Linda, CA, USA) connected to the facemask, measuring the flow in and out the infant. The signals were digitized at 200 Hz using the NewLifeBox-R physiological recording system (Advanced Life Diagnostics, Weener, Germany) and all signals were recorded by the NewLifeBox Neo-RSD computer system (Advanced Life Diagnostics, Weener, Germany) supported by Polybench physiological software (Applied Biosignals, Weener, Germany). Pulmochart software (Applied Biosignals, Weener, Germany) was used to calculate the mean airway pressure averaging the airway pressure between two inspiratory onsets.

Recordings started 1 min after birth, with the exception of infants who were delivered on the resuscitation table within the amniotic sac, as previously described by Mehler et al. (31) Opening of the amniotic sac was considered time of birth. Raw data were assessed on validity by the best clinical judgment of the researcher, as the signal IQ was not collected by the respiratory function monitor. In case of doubt, a second researcher assessed the data. Each presented value is calculated based on 60 measurements within a 30 s interval, if <10 measurements were available the mean was excluded from the analysis.

### Ethics

The local institutional Research Ethics Committee of the LUMC and the University Hospital of Cologne and the LUMC approved the study protocol and issued a statement of no objection for performing this research.

### Sample Size Calculation

Sample size was calculated based on the oxygen saturation of an independent sample. In the clinical trial of Dekker et al. (32) infants below 28 weeks of gestation received 5–8 cm H_2_O of CPAP according to the LUMC local protocol. The mean ± sd oxygen saturation in the first 7 min after birth was 68 ± 13%. To detect an absolute increase of 10% in SpO_2_ when using higher pressure levels, using a power of 80% and α = 0.05, 54 infants (28 in each group) where calculated to be required. Infants included in the clinical trial (32), thus included in the sample size calculation, were excluded in this study.

### Statistical Analyses

Data were analyzed using IBM SPSS Statistics version 23.0 (IBM Software, Chicago, Illinois, USA, 2016). Categorical data were analyzed using Related-Samples McNemar tests and are presented as *n* (%). Numerical data were assessed for normality by visual inspection of histograms. The data were analyzed by the Related-Samples Wilcoxon Signed Rank test and presented as median (IQR).

Physiological parameters were compared between groups over time using a linear mixed-effect regression model, accounting for the relation between multiple measurements of the same infant with a heterogeneous first-order autoregressive covariance structure on the residuals. Group, time and group^*^time interaction were included as fixed factors in the model. The model corrected for Apgar score ‘1 minute after birth. As all infants born at the University Hospital of Cologne were born by cesarean section, we were unable to correct for mode of delivery. Therefore, a sensitivity analysis was also performed including infants born by cesarean section only. The SpO_2_ and FiO_2_ variables were transformed using logit transformations: Ln(X/(1–X)) for SpO_2_ and Ln(79/100^*^(X−0.21)/(1–79/100^*^(X−0.21))) for FiO_2_, to ensure that the estimated values of both parameters remained between 0–100% and 21–100%, respectively, in the statistical model. Raw data is presented as median (IQR). *P*-values <0.05 were considered statistically significant and reported *p*-values are two sided.

## Results

**Figure 1:**
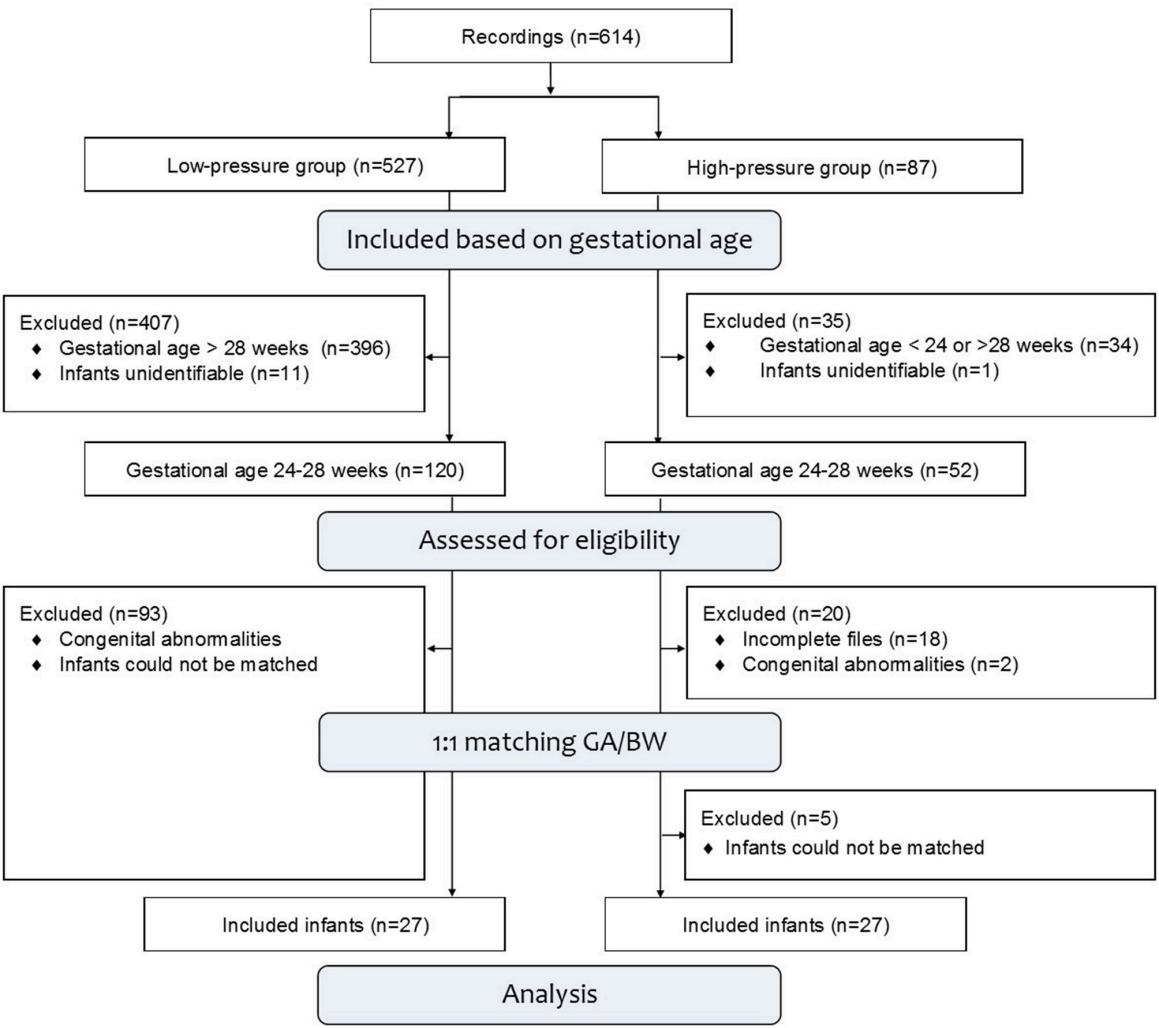
Study population. The LUMC and the University Hospital of Cologne stored 614 recordings, not including corrupt files. Recorded infants were assessed for eligibility and excluded based on gestational age, congenital abnormalities or unidentifiable video recordings. Infants who could not be matched were excluded as well as the remaining infants from the LUMC.

In total, 54 infants were included in this study. The University Hospital of Cologne and the LUMC stored 87 and 527 recordings of neonatal resuscitations. Twelve infants could not be identified and 429 infants were excluded based on gestation. One hundred and twenty five infants were excluded due to congenital abnormalities, incomplete files or because they could not be matched (Figure 1). The groups were similar in gestational age, birth weight, gender, application of steroids and tactile stimulation (yes or no) within the first 7 min after birth (Table 1). Mode of delivery (low- vs. high-pressure group; % cesarean section; 44 vs. 100%, *p* < 0.001) and Apgar score ‘1 min after birth (5 (3–7) vs. 6 (5–7), *p* = 0.048) were significantly different between groups. Nine infants of the high-pressure group were delivered within the amniotic sac, and for two infants it is unknown if they were delivered within the amniotic sac.

**Table 1:** Baseline characteristics.

	**Low-pressure group (*n* = 27)**	**High-pressure group (*n* = 27)**	***p*-value**
Gestational age (weeks)^a^	26 1/7 (24 6/7–27 3/7)	26 0/7 (24 5/7–27 2/7)	0.459
Birth weight (grams)^a^	827 (660–975)	750 (650–960)	0.156
Gender (% male)^b^	13 (48)	17 (63)	0.388
Antenatal corticosteroids (% started)^b^	25 (93)	24 (89)	1.000
Antenatal corticosteroids (% full dose)^b^	13 (52)^*^	18 (78)^*^	0.070
Mode of delivery (% cesarean section)^b^	12 (44)	27 (100)	< 0.001
Tactile stimulation^b^	17 (74)^*^	15 (60)^*^	0.508
Apgar score at 1 minute after birth^a^	5 (3–7)	6 (5–7)	0.026

*Data are presented as median (IQR) and n (%), p-values are presented of (a) Related-Samples Wilcoxon Signed Rank Test and (b) Related-Samples McNemar test*.

* *N < 27*.

**Figure 2:**
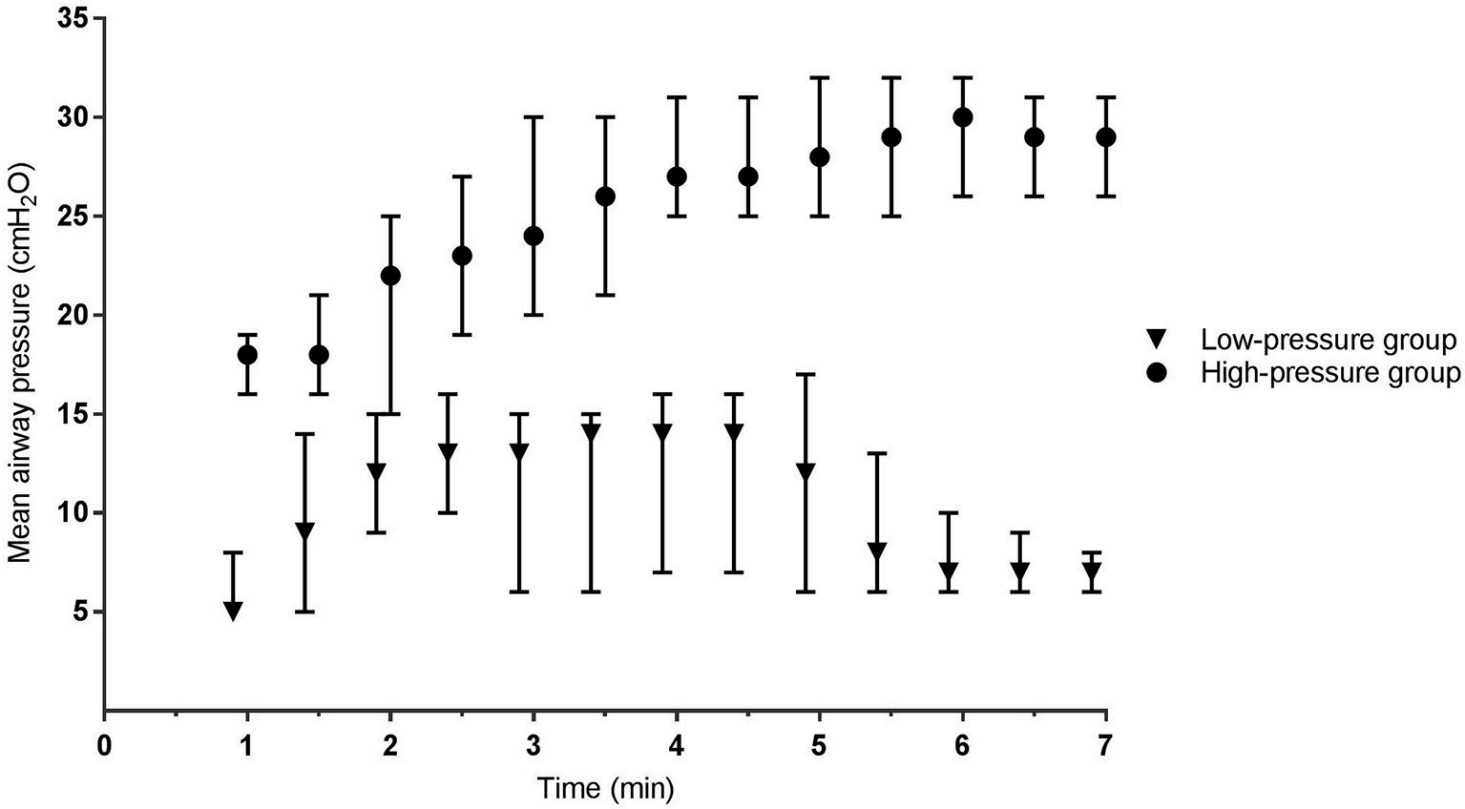
Mean airway pressure. Data is presented as median (IQR). Mean airway pressure is the average airway pressure between two inspiratory onsets. In the low-pressure group this includes CPAP, sustained inflations and positive pressure ventilation. In the high-pressure group CPAP was exclusively used.

**Figure 3:**
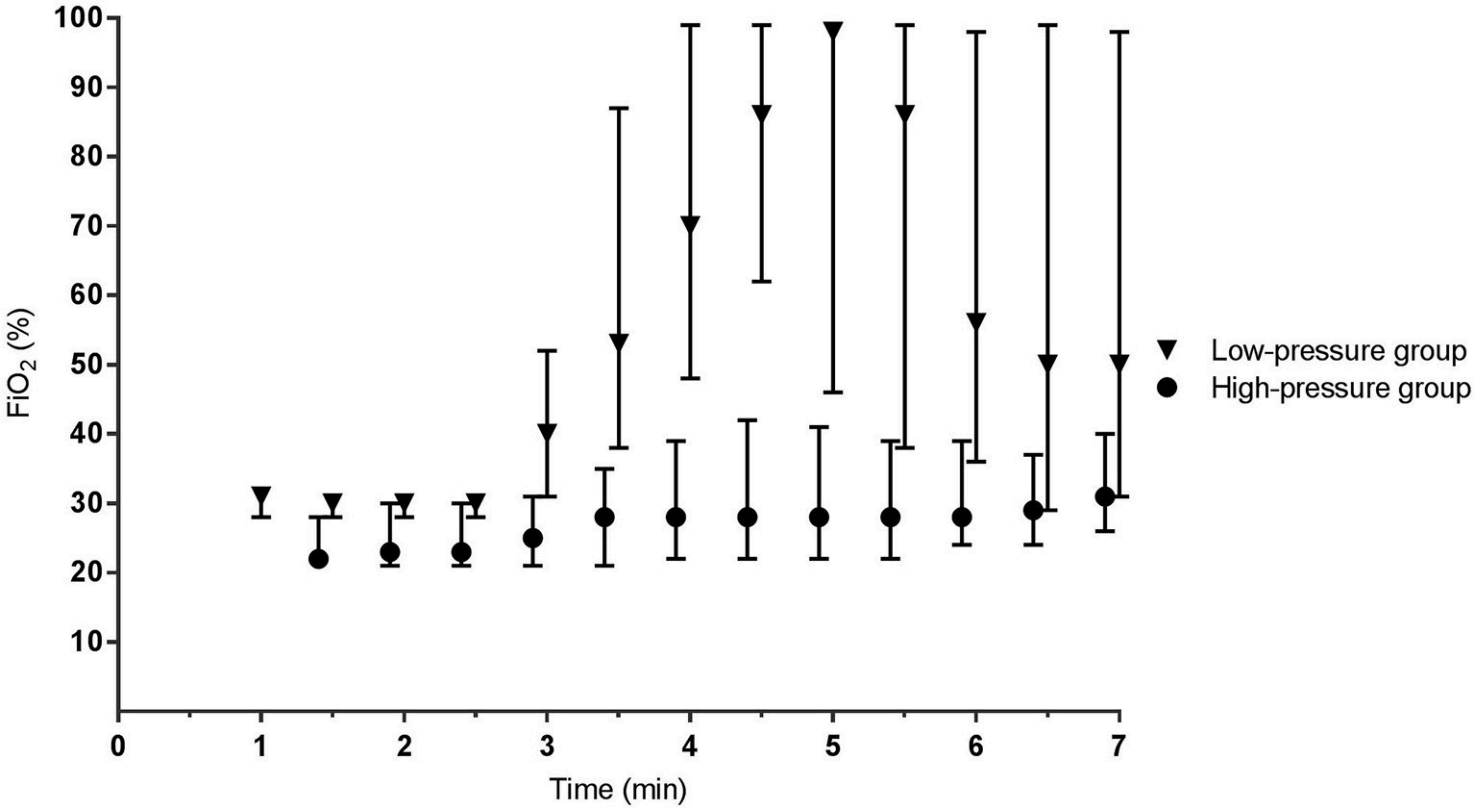
Fraction of inspired oxygen. Data is presented as median (IQR).

**Figure 4:**
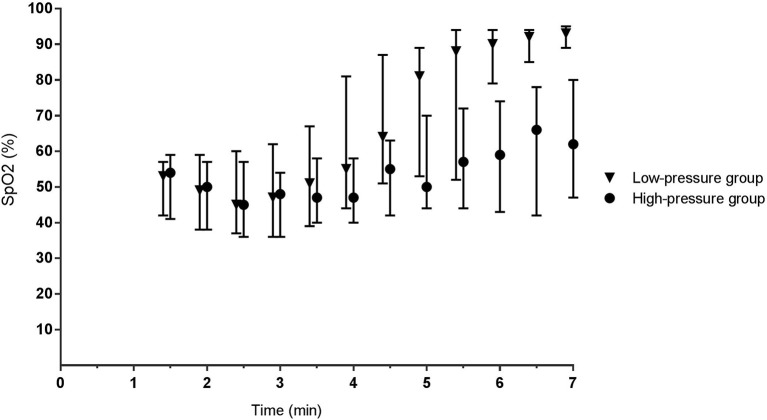
Oxygen saturation. Data is presented as median (IQR). For the high-pressure group, at 1.5 and 2 min, the mean of the high-pressure group is calculated based on *n* = 9 and *n* = 16, respectively. All other presented data is based on *n* ≥ 20.

**Figure 5:**
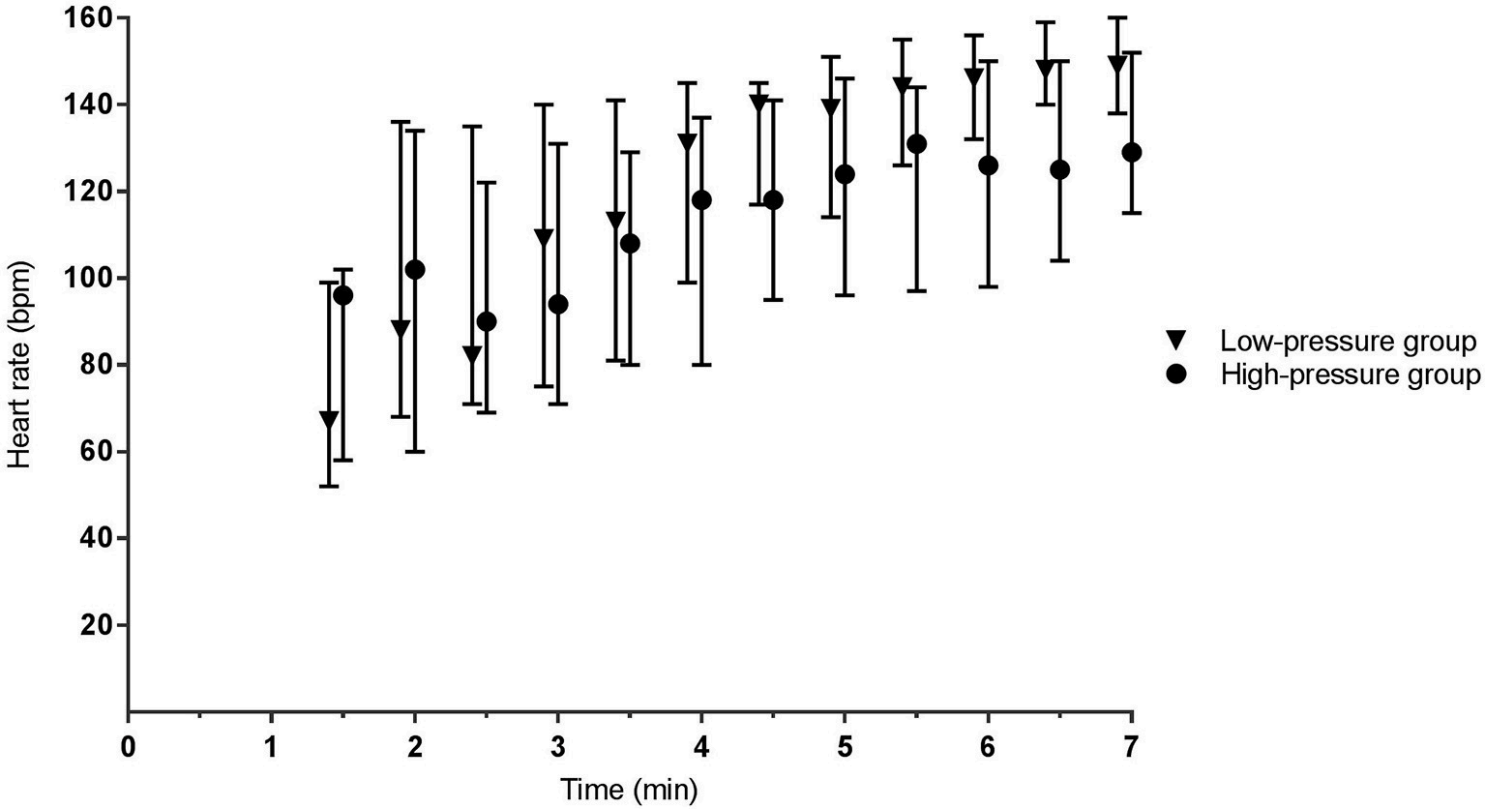
Heart rate. Data is presented as median (IQR). At 1.5 and 2 min, the mean of the high-pressure group is calculated based on *n* = 9 and *n* = 16, respectively. All other presented data is based on *n* ≥ 20.

**Figure 6:**
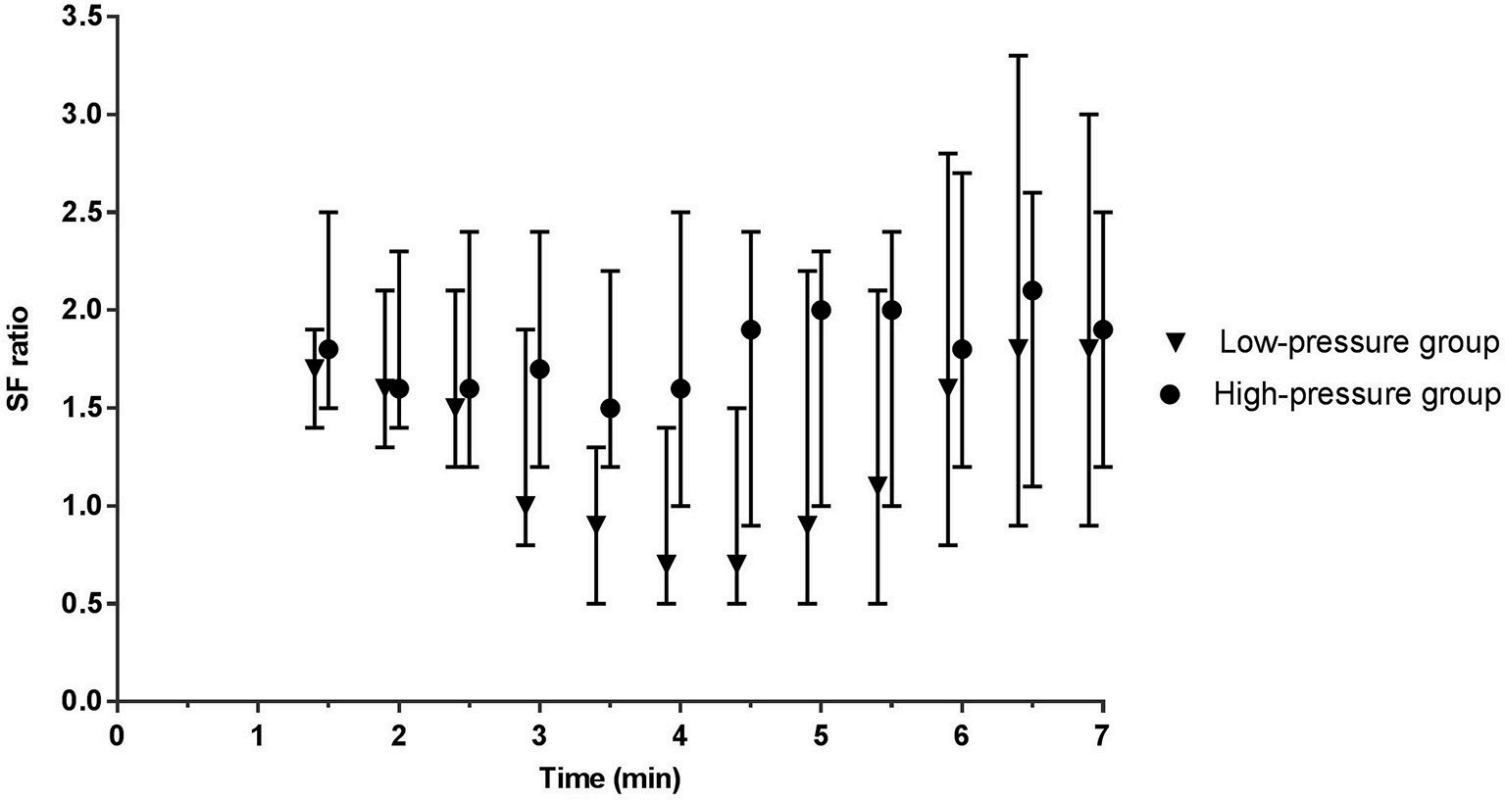
SF ratio. The SF ratio (SpO_2_/FiO_2_) represents gas exchange over the lungs and corrects the SpO_2_ for the given FiO_2_. Data is presented as median (IQR). At 1.5 and 2 min, the mean of the high-pressure group is calculated based on *n* = 9 and *n* = 16, respectively. All other presented data is based on *n* ≥ 20.

During phase I, the mean airway pressure [low- vs. high-pressure group 12 (6–15) vs. 19 (16–23) cm H_2_O, *p* < 0.001] (Figure 2) and FiO_2_ [0.30 (0.28–0.31) vs. 0.22 (0.21–0.30), *p* < 0.001] were different between groups (Figure 3). SpO_2_ [48 (38–59) vs. 49 (38–57)%, p = 0.759] (Figure 4), heart rate [79 (66–130) vs. 96 (62–120) bpm, *p* = 0.576] (Figure 5), and SpO_2_/FiO_2_ ratio [1.3 (0.8–2.0) vs. 1.8 (1.2–2.3), *p* = 0.348] (Figure 6) were similar between groups.

During phase II, the difference in mean airway pressure [8 (6–15) vs. 28 (24–31) cm H_2_O, *p* < 0.001] and FiO_2_ [0.62 (0.35–0.98) vs. 0.28 (0.22–0.38), *p* = 0.005] became more pronounced between groups. In the low-pressure group, caffeine was administrated to six infants at 4:45 (4:19–6:36) min. SpO_2_ [77 (50–92) vs. 53 (42–69)%, *p* < 0.001] was significantly higher in the low-pressure group, whereas heart rate [141 (114–151) vs. 122 (95–140) bpm, *p* = 0.293] and SpO_2_/FiO_2_ ratio were not different between groups [1.0 (0.6–2.1) vs. 1.8 (1.1–2.4), *p* = 0.483] (Figure 6).

The mean airway pressure of the low-pressure group exceeded the CPAP limit of 8 cm H_2_O due to contributions of sustained inflations and PPV (Figure 2). 20/27 (74%) infants received sustained inflations. Pressures for the first and second sustained inflations were 20 (20–22) and 24 (23–25) cm H_2_O, respectively. According to protocol, 16 infants received two sustained inflations. Three infants received only one sustained inflation, and one infant received three sustained inflations. 22/27 (82%) infants received PPV for 2:08 (1:19–3:01) min using positive inspiratory pressures of 26 (25–27) cm H_2_O.

Short-term clinical outcomes were comparable between the groups; only intubation rate during admission was significantly higher in the low-pressure group (70 vs. 30%, *p* = 0.013). The pneumothorax incidence (4 vs. 19%, *p* = 0.125) and occurrence of spontaneous intestinal (0 vs. 15%, *p* = 0.125) perforations during admission were not significantly higher in the high-pressure group (Table 2).

**Table 2:** Short-term clinical outcomes.

	**Low-pressure group (*n* = 27)**	**High-pressure group (*n* = 27)**	***p*-value**
Apgar score at 5 min after birth^a^	8 (6–9)	8 (7–8)	0.947
Intraventricular hemorrhage >grade 2 (%)^b^	3 (11)	2 (7)	1.000
Pneumothorax incidence < 72 h after birth (%)^b^	1 (4)	3 (11)	0.500
Pneumothorax incidence during hospitalization (%)^b^	1 (4)	5 (19)	0.125
Intubation rate < 72 h after birth (%)^b^	15 (56)	7 (26)	0.057
Intubation rate during hospitalization (%)^b^	19 (70)	8 (30)	0.013
Spontaneous intestinal perforation (%)^b^	0 (0)	4 (15)	0.125

*Data are presented as median (IQR) and n (%), p-values are presented (a) Related-Samples Wilcoxon Signed Rank Test and (b) Related-Samples McNemar test*.

## Discussion

In recent years, the focus of respiratory support has shifted toward a more gentle approach (6–8) using non-invasive CPAP and PPV, and room-air or an oxygen/air blend to commence resuscitation (9–11). Still many very preterm infants are hypoxic in the first minutes after birth and take some time to reach oxygen target values (23). Since the focus has shifted toward supporting spontaneous breathing (6), the use of CPAP strategies have gained much interest, but it is still unknown as to what CPAP level is best and how this interacts with different oxygen strategies. Despite numerous clinical trials and meta-analysis, there is also still insufficient data to recommend an oxygen strategy for very preterm infants (28–30). This retrospective matched-pairs study explored two respiratory support approaches in the delivery room for very preterm infants focusing on high CPAP levels or oxygen.

During phase I of the study, the two centers used statistically different mean airway pressures and FiO_2_ levels. The difference in FiO_2_ was the least during this part of the study period. Also, at this stage of lung aeration the available surface area for gas exchange is relatively low. We therefore concluded that the FiO_2_ had the least clinical impact and the main treatment during this part of the study period was pressure levels. In these first minutes after birth, the different pressure levels did not affect SpO_2_, heart rate and SpO_2_/FiO_2_ ratio. In contrast to these findings, preclinical studies comparing CPAP (17) or PEEP (13–16, 18) suggested that high-pressure levels improve oxygen saturation and uniformity of lung aeration. Theoretically, high-pressure levels increase the surface area of the alveoli, and thereby the gas exchange surface area, which increases the efficiency of oxygen exchange, leading to increased oxygen saturations. A reason for achieving similar physiological outcomes, despite the different airway pressures, could be the closure of the glottis and the inability of the pressure to be transmitted down into the lower airways. In previous preclinical studies, animals were intubated and mechanically ventilated, whereas the preterm infants in this study received non-invasive support. A recent animal study (19) highlighted that closure of the glottis directly after birth can prevent the transmission of PPV into the lower airways and whether it is closed or open is closely associated with the breathing pattern. Infants in this study were initially hypoxic, which likely suppressed breathing activity and caused the glottis to adduct (19). This could impede the delivery of respiratory support and explain the similar physiological outcomes while using different pressure levels.

During phase II, the FiO_2_ was increased in the low-pressure group, resulting in a significantly higher SpO_2_ in this group. It is interesting that the SpO_2_/FiO_2_ ratio was not statistically different, suggesting that the gas exchange potential was similar between groups. Reasons for different SpO_2_ but similar SpO_2_/FiO_2_ ratios could be that while higher airway pressures increased the surface area for gas exchange, it was insufficient to equal a higher oxygen gradient for O_2_ diffusion across the alveolocapillary border. Clearly, the high-pressure group could have achieved the same SpO_2_ levels if more supplemental oxygen was given, but the FiO_2_ required to achieve the same SpO_2_ would likely be less in this group.

Short-term clinical outcomes were statistically similar between groups with exception of the intubation rate. Different cut-off values for intubation for e.g., pH and FiO_2_ potentially contributed to this difference. In the low-pressure group the threshold for intubation was lower and infants were directly intubated when CPAP failed the infant's respiratory needs, whereas infants of the high-pressure group first received non-invasive bi-level positive airway pressure or high frequency oscillation before being intubated. The occurrence of spontaneous intestinal perforations and pneumothoraxes during admission tended to be higher in the high-pressure group. The difference was not statistically different, most likely due to the limited sample size. It remains unknown to what extent the high-pressure in the delivery room may increase the risks, as infants in the high-pressure group continued to receive higher pressures at the ward. However, preclinical studies in lambs (14, 15) have also recorded a high pneumothorax incidence after using high PEEP pressures in recruited lungs. It has been suggested that neonatal resuscitation should commence with higher pressures to facilitate lung aeration when the lungs are liquid-filled, airway resistance is high and the lungs are less compliant (33). Currently, higher airway pressures are already used in the delivery room for lung aeration when using sustained inflations and PPV; in the low-pressure group 74% of the infants received sustained inflations of 20 and 24 cm H_2_O and 81% of infants received PPV using inflation pressures of 26 cm H_2_O for 2:08 of the 7 min. Once lung liquid is replaced by air, airway resistance decreases and lung compliance increases (34, 35). Pressure levels should then be titrated down to reduce the risk of adverse events such as pneumothoraxes. If infants need more respiratory support, oxygen could increase the concentration gradient thereby driving oxygen transfusion over the lungs. By dynamic titration of CPAP and oxygen based on physiological parameters, each infant gets an individual approach suited for their conditions, while minimizing the risk of overexpansion and an overly high oxygen load. Until the optimal CPAP levels are examined extensively in preclinical settings, the use of high-pressure levels should carefully be considered.

The retrospective aspect of this study comes with some pitfalls. Most importantly, the protocols at the two sites had differences that we were unable to correct. The low-pressure group used the Neopuff™ T-piece Resuscitator to provide pressure support, whereas this was provided by the Benveniste valve™ in the high-pressure group. Although bench tests (36–38) imply that the Benveniste valve™ reduces the work of breathing, the clinical relevance of this finding is unknown. Infants were also positioned differently (supine vs. lateral on the right side), although no differences in oxygenation and heart rate were previously detected when comparing left-sided and supine positions (39). As the respiratory function monitor is mostly used in infants born by cesarean section at the University Hospital of Cologne, most infants in the high-pressure group were delivered by cesarean section. As these infants require more respiratory support (40, 41), we corrected for this difference in the statistics model. We were also unable to correct for other parameters that can affect cardiopulmonary function in the newborn (32, 42–44), including; time of cord clamping, cord milking, delivery within the amniotic sac and caffeine administration within 7 min of birth.

In summary, most very preterm infants need respiratory support to transition from fetal to newborn life and start pulmonary gas exchange. Theoretically, gas exchange can be improved by; (i) increasing the gas exchange surface area by using higher CPAP levels and (ii) increasing the gradient for oxygen diffusion by increasing the FiO_2_. In this retrospective study we did not see a difference in oxygen saturation using different pressure levels, which is possibly due to the closure of the glottis, preventing the pressure being transmitted down into the lower airways. The oxygen saturation only increased after increasing the FiO_2_. Until further preclinical trials examine the effect of high-pressure CPAP, we should be careful in administering high pressures to avoid pneumothoraxes.

## Author Contributions

TM performed the literature search, data collection, interpretation and analysis, and wrote the manuscript. AtP designed the structure of the article, helped with data interpretation, and writing and editing of the report. AO, JD, and SH were involved with the data interpretation. NvG was involved in data analysis and revised the content of the statistics paragraph. All authors contributed to the final draft by reviewing the manuscript. All authors approved the final version to be published and agree on the accountability for all aspects of the work.

### Conflict of Interest Statement

The authors declare that the research was conducted in the absence of any commercial or financial relationships that could be construed as a potential conflict of interest.

## Abbreviations

CPAP: Continuous positive airway pressure
FiO_2_: Fraction of inspired oxygen
LUMC: Leiden University Medical Center
PEEP: Positive end-expiratory pressure
PPV: Positive pressure ventilation
SpO_2_: Oxygen saturation.
